# Association of low HDL-c levels with severe symptoms and poor clinical prognosis in patients with severe fever and thrombocytopenia syndrome

**DOI:** 10.3389/fmicb.2023.1239420

**Published:** 2023-08-31

**Authors:** Taihong Huang, Yinyin Fan, Yanyan Xia, Xuejing Xu, Xinyue Chen, Hongling Ye, Yuxin Chen, Sen Wang

**Affiliations:** ^1^Department of Clinical Laboratory Medicine, Nanjing Drum Tower Hospital Clinical College of Nanjing Medical University, Nanjing, China; ^2^Department of Pancreatic Surgery, Nanjing Drum Tower Hospital Clinical College of Jiangsu University, Nanjing, China

**Keywords:** severe fever with thrombocytopenia syndrome, novel bunyavirus, HDL, APoAI, biomarker

## Abstract

**Background:**

Severe fever with thrombocytopenia syndrome (SFTS) is an acute infectious disease caused by a novel bunyavirus, characterized by high fever, thrombocytopenia, and multiple organ damage. Disturbances in lipid metabolism often occur during viral infections, but the changes and clinical significance of lipid profiles in SFTS patients remain unclear. This study aimed to investigate the alterations in lipid profiles and their clinical significance in SFTS patients.

**Methods:**

A total of 157 SFTS patients and 157 healthy controls were enrolled in this study. Serum lipid levels were collected and analyzed among different groups and prognosis categories. Receiver operating characteristic (ROC) curve analysis was performed to assess the ability of lipid levels in distinguishing between severe and mild cases, as well as surviving and non-surviving patients. Pearson correlation analysis was used to examine the associations between lipid levels and clinical laboratory parameters.

**Results:**

SFTS patients exhibited significantly lower levels of HDL-c, LDL-c, cholesterol, APoAI, and ApoB compared to healthy controls, while triglyceride levels were significantly higher. Serum HDL-c and ApoAI demonstrated good performance as indicators for distinguishing between survivors and non-survivors (AUC of 0.87 and 0.85, respectively). Multivariate regression analysis indicated that HDL-c independently acts as a protective factor in patients with SFTS. HDL-c levels showed decline in non-survivors but recovered in survivors. Moreover, HDL-c exhibited significant correlations with various clinical laboratory parameters (IL-6, CRP, AST, TT, APTT, PLT, ALB, and CD4).

**Conclusion:**

This study identified abnormalities in serum lipid metabolism among SFTS patients. HDL-c and ApoAI levels hold potential as biomarkers for distinguishing survivors from non-survivors. Additionally, HDL-c and ApoAI may serve as therapeutic targets for the management of SFTS patients.

## 1. Introduction

Severe fever with thrombocytopenia syndrome (SFTS) is an emerging infectious disease caused by a novel bunyavirus. Over the past few years, SFTS has garnered global attention due to its significant impact. The initial recognition and naming of SFTS occurred in 2009 when a patient presented with clinical manifestations of persistent high fever, general malaise, and thrombocytopenia (Yu et al., 2011). Since then, an increasing number of similar cases have been reported, particularly in the Asian region, notably in China, Japan, and South Korea (Yoshikawa et al., 2015; Yun et al., 2017). The cases of SFTS are primarily distributed in rural areas within mountainous and hilly regions, occurring sporadically. This disease is most prevalent during the summer, and the population is generally susceptible. SFTS virus (SFTSV) is an RNA virus with a single-stranded genome, primarily transmitted through tick bites (Ren et al., 2021). Ticks serve as the main vectors responsible for infecting hosts, including humans, livestock, and wild animals, thereby facilitating the transmission of SFTSV (Luo et al., 2015). The clinical presentation of SFTS primarily encompasses abrupt onset of high fever, fatigue, headache, muscle and joint pain, alongside notable thrombocytopenia. Patients frequently exhibit bleeding manifestations, including epistaxis, skin congestion, and gum bleeding. Severe cases may involve gastrointestinal bleeding, central nervous system symptoms, and multiple organ failure (Wang et al., 2022). Although the clinical manifestations of SFTS bear similarities to other viral hemorrhagic fevers, this disease demonstrates a more rapid progression and higher case fatality rate (Zhao et al., 2021). The underlying mechanisms of SFTSV infection remain incompletely elucidated, and regrettably, there is currently no specific therapy or vaccine available for combating SFTS. Consequently, precise evaluation of disease severity and accurate prediction of disease progression assume paramount importance.

Abnormal lipid metabolism is pivotal in the development and progression of a range of diseases, including cardiovascular disease, metabolic syndrome, and diabetes (Tenenbaum et al., 2006; Chen et al., 2022). High-density lipoprotein (HDL) stands as a key player in lipid metabolism, regulating cholesterol regulation and demonstrating noteworthy capacities in anti-infection, anti-inflammation, anti-apoptosis, and anti-oxidation functions (Grunfeld et al., 1999; Murphy et al., 2008). Viral infections have been found to impact lipid metabolism, leading to alterations in serum lipid concentrations, particularly cholesterol levels, among patients infected with human immunodeficiency virus (HIV) and hepatitis C virus (HCV) (Kulasegaram et al., 2005; Bassendine et al., 2013). In the case of SARS-CoV-2 infection, COVID-19 patients experience significant decreases in low-density lipoprotein cholesterol (LDL-C) and high-density lipoprotein cholesterol (HDL-C), with low levels of HDL-C being associated with a poorer prognosis (Sun et al., 2020; Masana et al., 2021). The understanding of lipid metabolism and its clinical implications in patients with SFTS is currently lacking. This study aimed to investigate the relationship between lipid levels and disease prognosis among SFTS patients.

## 2. Materials and methods

### 2.1. Patients and control participants

This study analyzed a total of 157 hospitalized patients who were diagnosed with fever and thrombocytopenia at the Department of Infectious Diseases in Nanjing Drum Tower Hospital between January 2021 and December 2022. This study was approved by the Institutional Review Board (IRB) of Nanjing Drum Tower Hospital (2022–238-02), Nanjing, China. All patients underwent testing for SFTSV RNA using real-time reverse transcription polymerase chain reaction, confirming SFTSV infection. The patient cohort comprised 80 males and 77 females, with an average age of 61.8 ± 11.1 years. Based on the prognosis of SFTS patients, they were categorized into two groups: survivors and non-survivors. Severe cases were defined as patients meeting any of the following criteria (Deng et al., 2013): multi-organ dysfunction, acute respiratory distress syndrome (ARDS), sepsis, disseminated intravascular coagulation (DIC), failure of one or more organs (such as heart failure, acute renal failure, or liver failure), infection-induced toxic shock, or death. A total of 157 individuals were recruited from the Physical Examination Center of Nanjing Drum Tower Hospital to serve as the control group. The healthy control group included 83 males and 74 females, with an average age of 60.7 ± 9.2 years. No significant variations were observed in terms of sex or age between the control group and the patient group. Standardized forms were utilized to gather clinical data from the electronic medical record system, encompassing demographic information, laboratory results, and clinical symptoms. The demographic and clinical characteristics of the study population are summarized in Table 1.

**Table 1:** Baseline characteristics for patients with SFTS.

Parameters	Survival	Non-survival	*p*	Mild symptoms	Severe symptoms	*p*
No.	129	28	–	101	56	–
Male/Female (n)	66/63	14/14	–	51/50	29/27	–
Age (years)	60.8 ± 11.4	66.5 ± 8.3	0.01	60.6 ± 11.6	64.1 ± 9.8	0.06
BMI (kg/m^2^)	23.6 ± 3.3	23.5 ± 3.4	0.94	23.6 ± 3.3	23.4 ± 3.3	0.90
Days of hospital stay	11.5 ± 6.5	7.2 ± 7.1	0.002	11.1 ± 6.8	10.1 ± 6.9	0.36
Time from onset to admission (days)	11.2 ± 9.3	10.7 ± 11.2	0.81	11.8 ± 10.4	9.8 ± 8.1	0.23
History *n* (%)						
Hypertension	42 (33%)	12 (43%)	0.29	35 (35%)	19 (34%)	0.93
Diabetes	10 (8%)	5 (18%)	0.09	10 (10%)	5 (9%)	0.84
Cardiovascular disease	51 (40%)	13 (46%)	0.50	31 (31%)	33 (59%)	0.0006
Cerebrovascular disease	31 (24%)	15 (54%)	0.001	21 (21%)	25 (45%)	0.002
kidney disease	38 (29%)	17 (61%)	0.002	28 (28%)	27 (48%)	0.003
Liver Disease	108 (84%)	27 (96%)	0.08	84 (83%)	51 (91%)	0.17
Cancer	3 (2%)	0 (0%)	0.42	3 (3%)	0 (0%)	0.19
Laboratory findings						
WBC (×10^9^/L)	5.4 ± 4.2	6.1 ± 4.2	0.41	5.4 ± 4.1	5.7 ± 4.5	0.61
NEU (×10^9^/L)	3.9 ± 4.3	3.9 ± 3.8	0.95	3.9 ± 4.3	3.9 ± 4.2	0.96
LYM (×10^9^/L)	1.2 ± 1.1	1.0 ± 0.7	0.35	1.2 ± 1.2	1.0 ± 0.6	0.26
HGB (g/L)	122.3 ± 22.0	124.6 ± 22.1	0.61	122.4 ± 22.5	123.3 ± 21.1	0.79
PLT (×10^9^/L)	104.6 ± 88.1	52.0 ± 48.7	0.003	108.0 ± 86.5	72.5 ± 77.1	0.01
ALT (U/L)	114.8 ± 188.6	96.2 ± 80.8	0.61	113.2 ± 206.2	108.4 ± 93.6	0.87
AST (U/L)	177.1 ± 194.8	425.6 ± 363.6	<0.0001	167.0 ± 179.6	319.6 ± 324.3	0.0002
LDH (U/L)	711.8 ± 552.8	2096 ± 2025	<0.0001	723.2 ± 579.8	1,370 ± 1,610	0.0004
ALB (g/L)	33.9 ± 4.3	29.2 ± 3.8	<0.0001	34.5 ± 4.3	30.7 ± 4.1	<0.0001
GLB (g/L)	28.8 ± 7.8	27.9 ± 6.1	0.56	28.2 ± 7.7	29.5 ± 7.2	0.29
CRP (mg/L)	12.1 ± 22.2	34.1 ± 40.9	0.0001	9.7 ± 16.6	27.3 ± 38.3	0.0001
IL-6 (pg/ml)	30.3 ± 63.8	878.1 ± 2,335	0.02	21.6 ± 23.9	542.4 ± 1822	0.12

The data was presented as the mean ± standard deviation (SD). BMI (Body Mass Index), WBC (White Blood Cell), NEU (Neutrophil), LYM (LYM), HGB (Hemoglobin), PLT (Platelets), ALT (Alanine Aminotransferase), AST (Aspartate Aminotransferase), LDH (Lactate Dehydrogenase), ALB (Albumin), GLB (Globulin), CRP (C-Reactive Protein).

### 2.2. Detection and collection of clinical parameters

Cholesterol, high-density lipoprotein cholesterol (HDL-C), low-density lipoprotein cholesterol (LDL-C), apolipoprotein AI (ApoAI), apolipoprotein B (ApoB), immunoglobulins (IgG, IgA, IgM), albumin (ALB), C-reactive protein (CRP), complement proteins C3 and C4, alanine aminotransferase (ALT), and aspartate aminotransferase (AST) levels, lactate dehydrogenase (LDH) along with other biochemical indicators, were measured using a biochemical analyzer (Beckman AU5400, Germany). CRP, IgG, IgA, IgM, C3, C4 were detected using the immunoturbidimetric method. Prothrombin time (PT), activated partial thromboplastin time (APTT), Fibrinogen and D-dimer were measured using Sysmex CS-5100 automated coagulation analyzer. Blood cell counts, hemoglobin, and other hematological parameters were determined using an automated hematology analyzer (Sysmex Corporation, Japan). All data were extracted from the hospital’s laboratory information system and medical record system.

### 2.3. Statistical analysis

Statistical comparisons between SFTS patients and healthy controls were conducted using Student’s t-test. Differences were deemed statistically significant if the *p*-value was less than 0.05 (*p* < 0.05). All data measurements are presented as mean ± standard deviation (m ± S). Pearson correlation analysis was employed to explore the relationships among clinical parameters, with correlations considered significant if the r value was greater than 0.2 and the p-value was less than 0.05 (*r* > 0.2, *p* < 0.05). Following univariate analysis, variables exhibiting statistical significance were chosen for subsequent multivariate logistic regression analysis. The outcomes of both univariate and multivariate regression analyses are presented as hazard ratios (HR) along with corresponding 95% confidence intervals (CI). A significance level of *p* < 0.05 was employed to determine statistical significance. Statistical analysis and data visualization were performed using SPSS 29.0 and GraphPad Prism 8.3 software.

## 3. Results

### 3.1. Clinical characteristics of the patients

This study included 157 SFTS patients, comprising 129 (82%) survivors and 28 (18%) non-survivors, with 56 (36%) classified as severe cases and 101 (64%) as mild cases. A comprehensive overview of the clinical characteristics of these patients is presented in Table 1. In this study, it was observed that non-survivors were characterized by advanced age compared to survivors. Additionally, the non-survivor group exhibited a significantly shorter duration of hospital stay compared to the survivor group. A higher proportion of non-survivors had a medical history of cerebrovascular disease and renal disease, in contrast to the survivor group. However, no significant differences were found between the survivor and non-survivor groups regarding the presence of hypertension and diabetes. In terms of clinical laboratory parameters, significant differences were noted in platelet count (PLT), AST, LDH, ALB, and CRP levels between the survivor group and the non-survivor group, as well as between the mild disease group and the severe disease group.

### 3.2. Altered lipid metabolism patterns observed in SFTS patients

**Figure 1:**
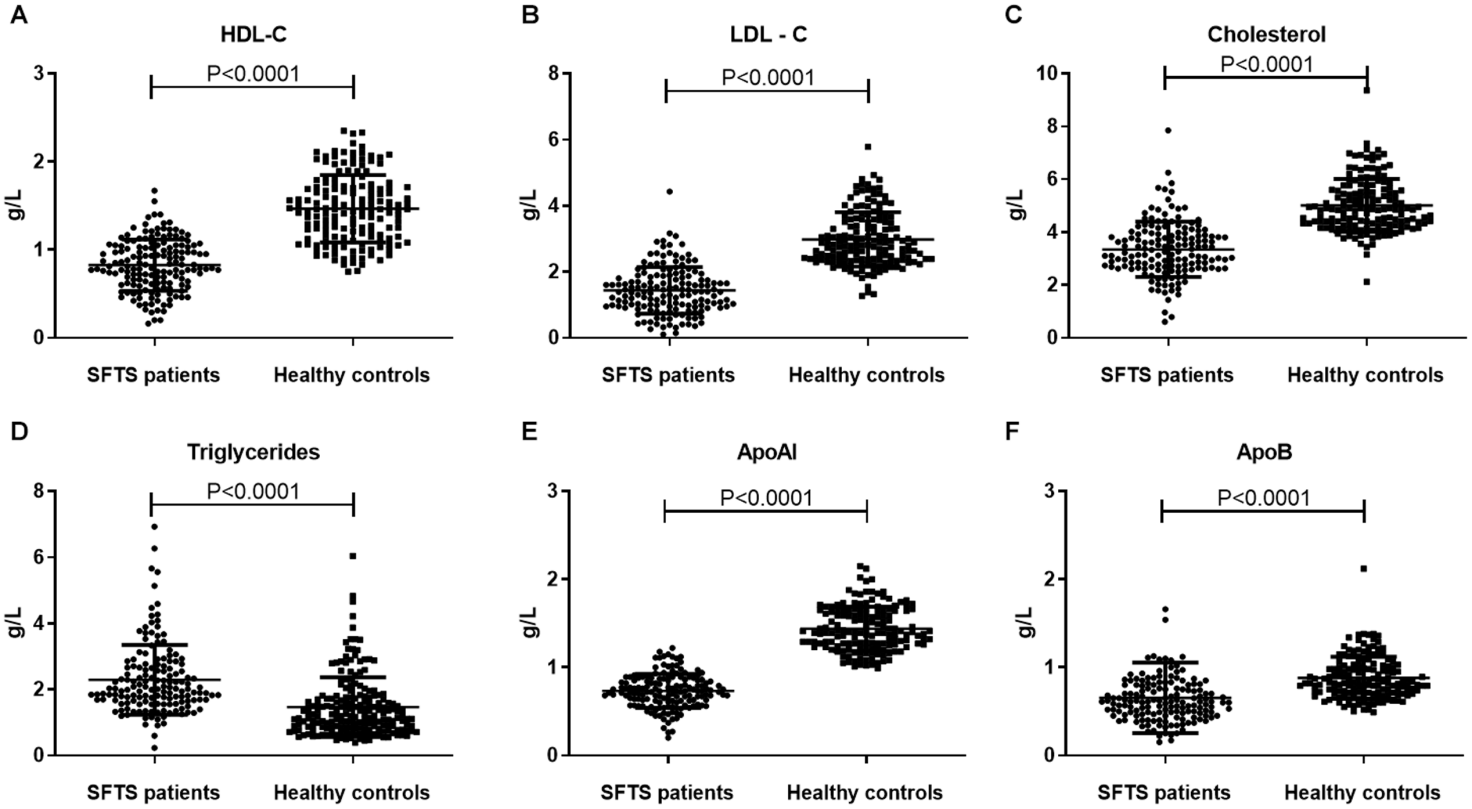
Comparative analysis of lipid profiles between SFTS patients and healthy controls. Comparison of serum lipid profiles (HDL-c, LDL-c, Cholesterol, Triglycerides, APoAI, and ApoB) between SFTS patients (*n* = 157) and healthy controls (*n* = 157) **(A–F)**. Data are expressed using mean ± standard deviation.

To investigate the levels and clinical significance of lipids in SFTS patients, we conducted a comprehensive analysis of serum lipids including HDL-c, LDL-c, cholesterol, triglycerides, APoAI, and ApoB in both SFTS patients and healthy controls. Our findings reveal substantial deviations in lipid profiles among SFTS patients compared to healthy controls. Specifically, we observed significant reductions in HDL-c, LDL-c, cholesterol, APoAI, and ApoB levels in the serum of SFTS patients. Conversely, triglyceride levels were significantly elevated (Figure 1). These alterations in lipid metabolism unequivocally signify a severe disturbance in the patient’s lipid profile following SFTSV infection.

### 3.3. Significant decrease in HDL-c levels observed in severe and non-survivor SFTS patients

**Figure 2:**
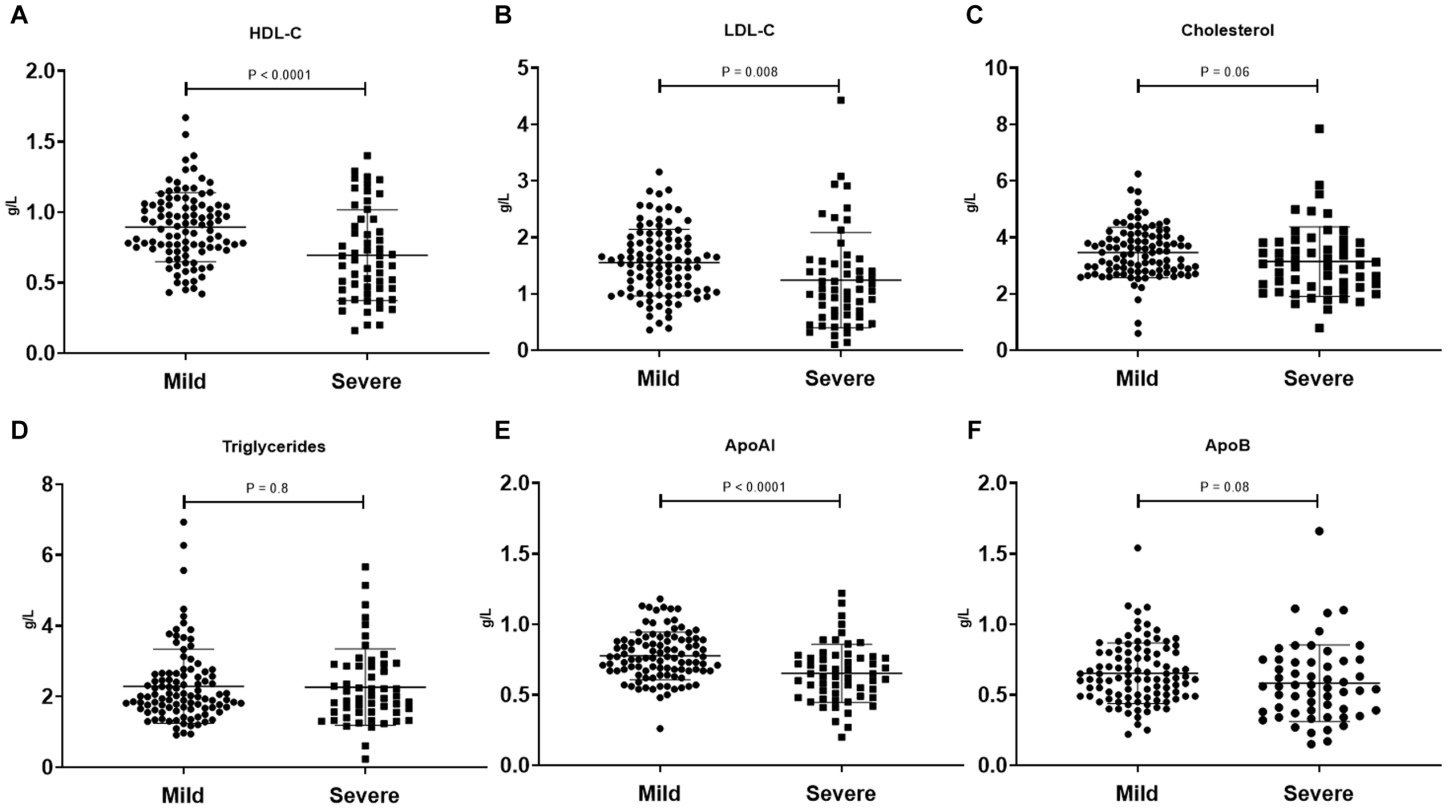
Comparison of serum lipid profiles (HDL-c, LDL-c, Cholesterol, Triglycerides, APoAI, and ApoB) between mild SFTS patients (*n* = 101) and severe SFTS patients (*n* = 56) **(A–F)**. Data are expressed using mean ± standard deviation.

To investigate the clinical significance of lipid metabolism-related indicators in SFTS patients, we conducted further analysis to compare lipid levels between severe and mild patients, as well as between survivors and non-survivors. Our findings reveal distinct patterns in lipid profiles within these patient groups. In severe patients, both HDL-c and LDL-c levels were significantly lower compared to those in mild patients, with the most noticeable decrease observed in HDL-c levels. Correspondingly, the main apolipoprotein of HDL, APoAI, also exhibited a significant decrease in critically ill patients. Conversely, no significant differences were observed in cholesterol, triglycerides, and APoB levels between the two groups (Figure 2). Additionally, we examined lipid levels in surviving patients and non-surviving patients. The results indicate significant reductions in HDL-c, LDL-c, cholesterol, APoAI, and APoB levels among non-surviving patients, while no significant difference was observed in triglyceride levels between the two groups (Supplementary Figure S1).

### 3.4. Prognostic potential of lipid metabolism parameters in predicting SFTS patient outcomes

**Figure 3:**
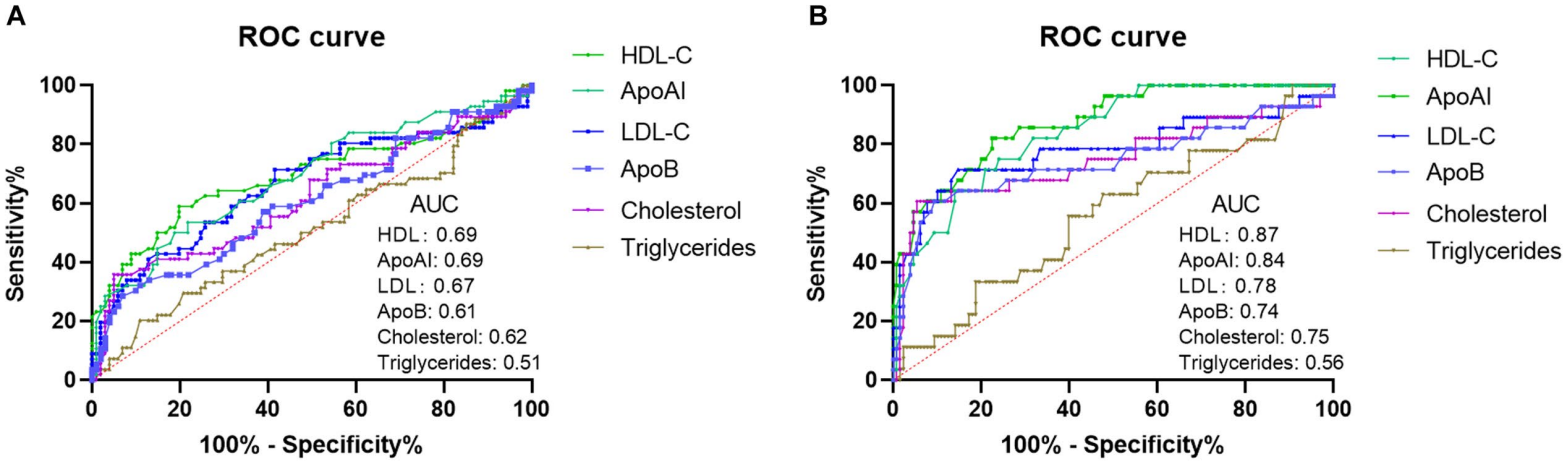
Analysis of ROC curves to assess the discriminatory ability of lipid profiles (HDL-c, LDL-c, Cholesterol, Triglycerides, APoAI, and ApoB) in distinguishing mild SFTS patients from severe SFTS patients **(A)**. Analysis of ROC curves to assess the ability of lipid profiles in distinguishing SFTS survivors from SFTS non-survivors **(B)**.

To investigate the predictive performance of lipid metabolism parameters as biomarkers for SFTS prognosis, we conducted ROC curve analysis to assess their ability to distinguish between severe and mild cases, as well as predict mortality versus survival. The results indicated that the effectiveness of lipid metabolism parameters in differentiating between severe and mild conditions was not remarkable. Among these parameters, HDL-c and ApoAI exhibited the highest performance, with an area under the curve (AUC) of 0.69 (Figure 3A). However, when used as markers to predict survival and non-survival, HDL-c and ApoAI demonstrated greater discriminatory ability, with AUCs of 0.87 and 0.84, respectively (Figure 3B). This suggests that HDL is the most suitable biomarker for assessing the prognosis of SFTS patients among the lipid metabolism parameters. Using a cutoff value of 0.71, it achieves a sensitivity of 82.1% and specificity of 77.5%.

### 3.5. Analyzing prognostic factors for poor outcomes in SFTS patients

We further evaluated the laboratory parameters influencing the poor prognosis in SFTS patients using univariate and multivariate regression analysis. As shown in Table 2, the univariate regression analysis revealed significant associations between outcomes in SFTS patients and Age, HDL-c, PLT, AST, LDH, Cholesterol, LDL-c, CRP, PT, APTT, Fibrinogen, D-dimer, and Albumin. Subsequently, these factors were included in the multivariate regression analysis, which identified HDL-c as an independent protective factor for the prognosis of SFTS patients.

**Table 2:** Univariate and multivariate regression analysis of laboratory parameters in SFTS patients and their prognostic implications.

Laboratory parameters	Univariable analysis		Multivariable analysis		HR (95% Cl)	*p* value	HR (95% Cl)	*p* value
Age	1.053 (1.010–1.098)	0.015	1.050 (0.962–1.146)	0.277
HDL-c	0.000 (0.000–0.009)	<0.001	0.000 (0.000–0.008)	0.007
PLT	0.984 (0.972–0,995)	0.005	0.983 (0.955–1.011)	0.220
AST	1.003 (1.002–1.005)	<0.001	0.998 (0.988–1.007)	0.631
LDH	1.001 (1.001–1.002)	<0.001	1.000 (0.998–1.002)	0.995
Cholesterol	0.434 (0.263–0.715)	0.001	2.052 (0.361–11.652)	0.417
LDL-c	0.204 (0.089–0.469)	<0.001	0.168 (0.005–5.271)	0.311
CRP	1.022 (1.009–1.036)	0.001	0.971 (0.933–1.010)	0.144
PT	2.798 (1.742–4.495)	<0.001	2.826 (0.729–10.963)	0.133
APTT	1.131 (1.080–1.185)	<0.001	1.034 (0.952–1.124)	0.425
Fibrinogen	0.497 (0.285–0.868)	0.014	1.719 (0.481–6.138)	0.404
D-dimer	1.15 (1.062–1.245)	<0.001	1.104 (0.971–1.254)	0.130
Albumin	0.744 (0.656–0.843)	<0.001	0.907 (0.687–1.198)	0.493

HR (hazard ratio), HDL-c (high-density lipoprotein cholesterol), PLT (Platelets), AST (Aspartate Aminotransferase), LDH (Lactate Dehydrogenase), LDL-c (high-density lipoprotein cholesterol), CRP (C-Reactive Protein), PT (Prothrombin time), APTT (activated partial thromboplastin time).

### 3.6. Dynamic changes in HDL-c levels among non-survivors and recovered patients

**Figure 4:**
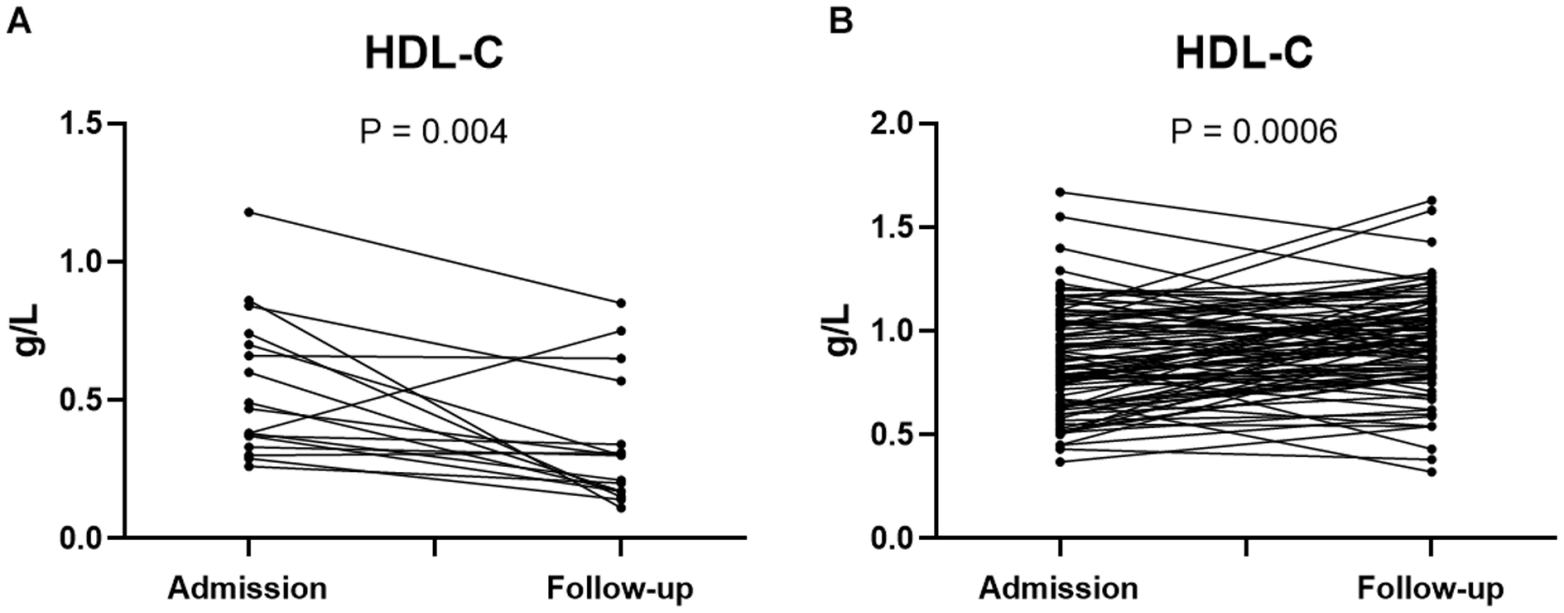
Dynamic changes of HDL-c level in admission and follow-up of SFTS non-survivors (*n* = 17) **(A)**. Dynamic changes of HDL-c level in SFTS surviving patients at admission and follow-up (*n* = 89) **(B)**. Statistical analysis using paired *T* test. Exclusion of patients with single HDL-c test from the analysis.

To further explore the association between HDL-c levels and the prognosis of SFTS patients, we examined the dynamic changes of HDL-c levels in both non-survivors and survivors. Among the non-survivor group, the study findings revealed that HDL-c levels in most non-surviving patients exhibited a decline as the disease progressed, with follow-up HDL-c levels significantly lower than the admission levels (Figure 4A). Conversely, within the survivor group, a notable rise in HDL-c levels was observed as the patients’ condition improved, with statistically significant results (Figure 4B). In addition, we also dynamically analyzed the HDL-c levels of patients in the survivor group and non-survivor group according to the days after admission. The results showed that the level of HDL-c in the non-survivor group continued to decline after hospitalization (Supplementary Figure S2).

### 3.7. Correlation analysis between HDL-c levels and clinical laboratory parameters

**Figure 5:**
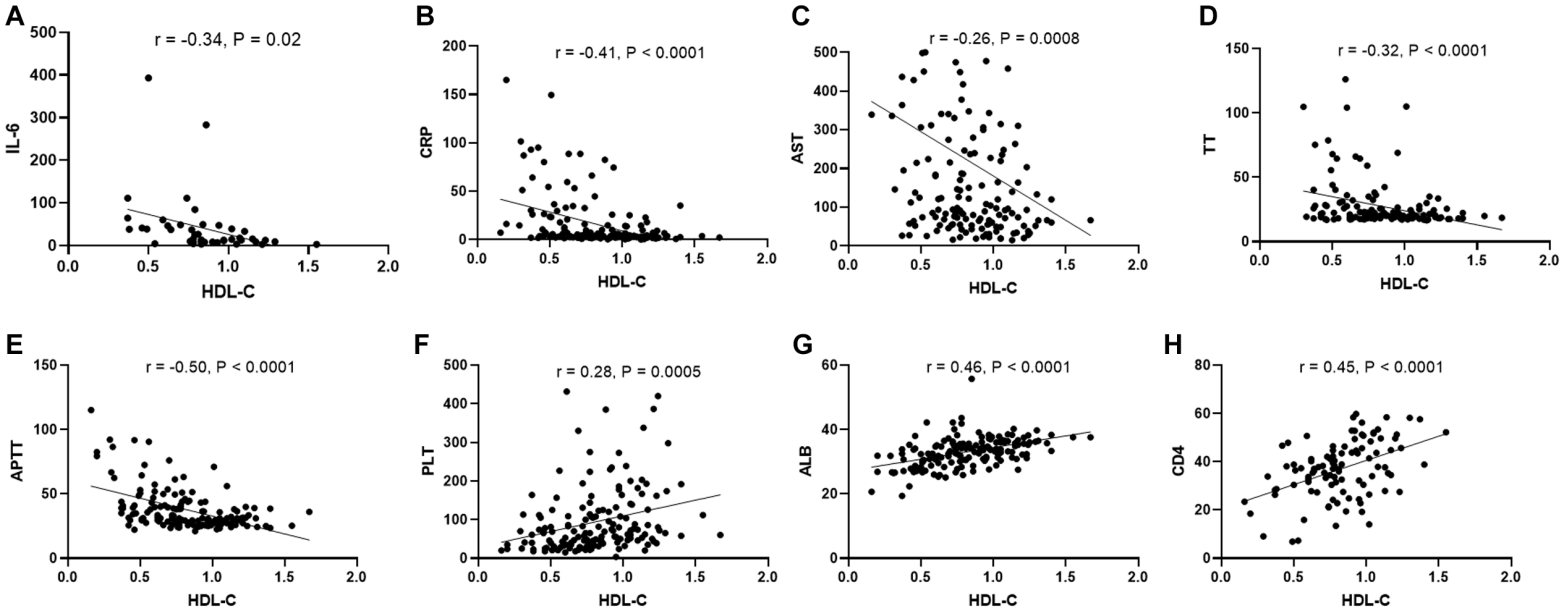
Correlation between HDL-c level and other laboratory parameters in SFTS patients. Correlation analysis of serum HDL-c level with IL-6, CRP, AST, TT, APTT，PLT, ALB, and CD4 counts in SFTS patients, respectively **(A–H)**.

During viral infections, several clinical laboratory parameters undergo significant changes, which are believed to be correlated with disease severity. To further investigate the clinical significance of HDL-c in SFTS patients, we conducted an in-depth analysis to explore the correlation between HDL-c levels and key clinical laboratory parameters. The results revealed a significant negative correlation between HDL-c and IL-6, CRP, AST, TT, APTT (Figures 5A–E), while demonstrating a significant positive correlation with PLT, ALB, and CD4 counts (Figures 5F–H). These findings provide additional evidence supporting the correlation between HDL-c levels and disease severity in SFTS patients.

## 4. Discussion

Various viral infections, including dengue virus, HIV, and SARS-CoV-2, have been associated with significant alterations in lipid profiles, which are considered prognostic indicators of disease (Maggi et al., 2017; Lima et al., 2019; Rezaei et al., 2022). In COVID-19 patients, LDL-C, HDL-C, and cholesterol levels were found to be decreased, particularly in severe and critically ill cases. Furthermore, among ICU-hospitalized COVID-19 patients, it has been observed that decreased HDL levels are linked to higher mortality rates (Wang et al., 2021).

The level and clinical significance of lipid profiles in SFTS patients have received limited attention. This study revealed significant disturbances in lipid metabolism hospitalized SFTS patients. Notably, LDL-c, HDL-c, and total cholesterol levels were significantly reduced, while triglyceride levels were significantly increased. These findings resemble the lipid metabolism disruptions observed in patients with HIV and COVID-19. Among critically ill patients, the most notable decrease was observed in HDL-c levels, and ROC curve analysis identified HDL-c as the most efficient marker in distinguishing non-survivors from survivors. Consequently, our research primarily focused on investigating HDL. We found a higher prevalence of cerebrovascular disease in severe or non-survivor patients, potentially due to immune and blood coagulation imbalances that worsen the condition. Univariate analysis linked age, HDL-c, PLT, and AST to patient prognosis. However, multivariate analysis, excluding other factors, highlighted HDL-c as an independent protective factor consistently associated with patient prognosis. We conducted a dynamic analysis of patients’ HDL-c levels. Among non-surviving patients, HDL-c levels did not increase but rather exhibited a decreasing trend as hospitalization time progressed. While there were no statistically significant differences between each time point, this might be attributed to the fact that not all patients underwent multiple HDL-c tests. Additionally, variations in the duration of hospital stays could have led to some mutual offsetting in HDL-c levels calculated based on days since admission. Our paired analysis, focusing on admission and follow-up as two time points, revealed a significant increase in HDL-c levels among survivors, whereas HDL-c levels in non-survivors notably decreased. Furthermore, HDL-c displayed significant correlations with several important clinical laboratory parameters, providing insights into the disease severity. Therefore, we propose that HDL-c holds potential as a prognostic marker for assessing the prognosis of SFTS patients.

HDL possesses multiple functions that contribute to its protective effects. One of its main functions is reverse cholesterol transport, where the protein ApoAI binds with free cholesterol in tissue cells and transports it to the liver, thus reducing overall cholesterol levels and delaying the onset and progression of coronary heart disease (Tosheska Trajkovska and Topuzovska, 2017). Furthermore, HDL has been shown to prevent systemic endotoxemia by binding and neutralizing lipopolysaccharide (LPS), serving as a crucial mechanism for its antibacterial effect (Parker et al., 1995). HDL also exerts anti-inflammatory effects by downregulating Toll-like receptor (TLR)-induced pro-inflammatory cytokines through the transcriptional regulator activating transcription factor 3 (ATF3) (de Nardo et al., 2014). Additionally, HDL exhibits antioxidant and anti-apoptotic properties (Nofer et al., 2001; Robbesyn et al., 2003). Given these functions, HDL can provide protective benefits for patients. In SFTS patients, we observed lower levels of HDL in severe and non-survivor patients, whereas mild and recovered patients exhibited higher HDL-c levels. Notably, HDL-c demonstrated a significant negative correlation with inflammatory indicators such as IL-6, CRP, and AST. These findings further suggest that HDL may play a protective role in SFTS patients.

The decrease in HDL-c levels during infection can be attributed to several mechanisms. Studies have indicated that pro-inflammatory cytokines like IL-6 can directly inhibit the activity of apolipoprotein synthase, leading to a reduction in HDL-C and apoA-1 production (Pirillo et al., 2015). Since HDL-c exerts anti-inflammatory effects, the deficiency of HDL-C exacerbates cytokine overproduction and further depletion of HDL-C. Inflammatory cytokines, such as TNF, can also decrease the activity of LCAT, resulting in reduced cholesterol lipid accumulation within HDL (Ly et al., 1995). Moreover, the downregulation of ABCA1 and ABCG1 by endotoxins can contribute to decreased HDL cholesterol levels (Schmitz and Langmann, 2005).

Additionally, in SFTS patients, it is noteworthy that while cholesterol levels significantly decreased, triglyceride levels were significantly higher compared to healthy controls, consistent with observations in COVID-19 patients. This phenomenon may be attributed to increased hepatic VLDL production and secretion during infection, which stimulates triglyceride synthesis (Feingold et al., 1992). Simultaneously, the infection and inflammation may inhibit the activity of lipoprotein lipase, leading to reduced clearance of triglycerides.

In addition to its potential as a prognostic biomarker for SFTS patients, this study also introduces a novel treatment concept. Studies have indicated that Omega-3 polyunsaturated fatty acids (PUFA) can improve lipid metabolism by reducing triglyceride levels and increasing HDL levels, while also reducing inflammatory responses (Ni et al., 2020). This approach has shown positive effects in the treatment of COVID-19 (Sorokin et al., 2020; Faguer et al., 2022). Furthermore, the use of statins to increase HDL levels is also a current research focus in the context of COVID-19 (Vahedian-Azimi et al., 2021). Presently, treatment options for SFTS patients are limited. Taking into account the potential protective role of HDL in SFTS patients, increasing HDL levels could potentially offer novel therapeutic strategies for managing SFTS. Of course, this requires further investigation into the biological mechanisms and treatment effects, through additional animal experiments and clinical trial data. This research direction is also highly meaningful.

In conclusion, this study revealed significant abnormalities in the serum lipid profile of SFTS patients, with a notable decrease in HDL-c levels observed in severe and deceased patients. HDL-c emerges as a potential biomarker for predicting poor prognosis in SFTS patients. These findings suggest that elevating HDL levels could be a novel therapeutic strategy for the treatment of SFTS patients.

## Data availability statement

The raw data supporting the conclusions of this article will be made available by the authors, without undue reservation.

## Ethics statement

The studies involving humans were approved by Institutional Review Board (IRB) of Nanjing Drum Tower Hospital. The studies were conducted in accordance with the local legislation and institutional requirements. Written informed consent for participation was not required from the participants or the participants' legal guardians/next of kin in accordance with the national legislation and institutional requirements.

## Author contributions

SW, YC, and TH contributed to the design of the study and supervised the scientific work. YF, YX, XX, XC, and HY contributed to the analysis and interpretation of the data. TH and YF drafted the manuscript and SW revised the manuscript. All authors contributed to the article and approved the submitted version.

## Funding

This work was supported by grants from Nanjing Medical Science and technique Development Foundation (QRX17142, YKK21066), Clinical Trials from the Affiliated Drum Tower Hospital, Medical School of Nanjing University (2022-LCYJ-PY-40). The funders had no role in the study design, data collection and analysis, decision to publish, or preparation of the manuscript.

## Conflict of interest

The authors declare that the research was conducted in the absence of any commercial or financial relationships that could be construed as a potential conflict of interest.

## Publisher’s note

All claims expressed in this article are solely those of the authors and do not necessarily represent those of their affiliated organizations, or those of the publisher, the editors and the reviewers. Any product that may be evaluated in this article, or claim that may be made by its manufacturer, is not guaranteed or endorsed by the publisher.
