# Supplementary material for: Association of low HDL-c levels with severe symptoms and poor clinical prognosis in patients with severe fever and thrombocytopenia syndrome
Source: Front Microbiol. 2023 Aug 31;14:1239420. doi: 10.3389/fmicb.2023.1239420 (PMC10501784; doi:10.3389/fmicb.2023.1239420)
Supplement: Supplementary file 1 [file Data_Sheet_1.pdf]

Association of Low HDL-c Levels with Severe Symptoms and Poor Clinical Prognosis in Patients with Severe Fever and Thrombocytopenia Syndrome

Taihong Huang[1, †], Yinyin Fan[2, †], Yanyan Xia[1], Xuejing Xu[1], Xinyue Chen[1], Hongling Ye[1], Yuxin Chen[1,*], Sen Wang[1,*]

[1] Department of Clinical Laboratory Medicine, Nanjing Drum Tower Hospital Clinical College of Nanjing Medical University, Nanjing, China

[2] Department of Pancreatic Surgery, Nanjing Drum Tower Hospital Clinical College of Jiangsu University, Nanjing, China

*Corresponding author:
Sen Wang, Email: njwangsen@163.com
Yuxin Chen, Email: yuxin.chen@nju.edu.cn

[†] Taihong Huang and Yinyin Fan contributed equally to this work.

Figure S1

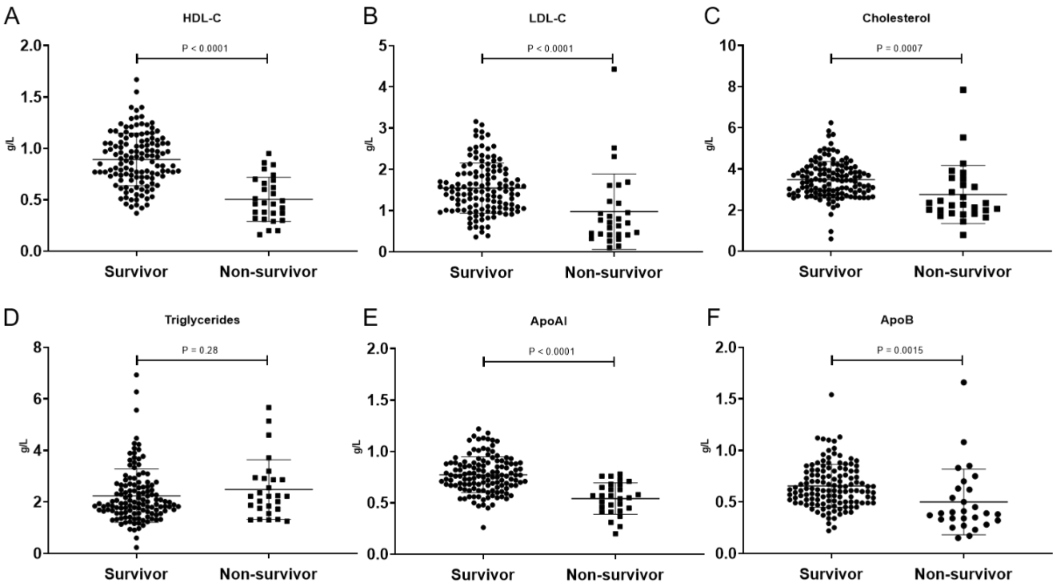

Figure S1. Comparison of serum lipid profiles (HDL-c, LDL-c, Cholesterol, Triglycerides, APoAI, and ApoB) between survivor (n = 129) and non-survivor SFTS patients (n = 28) (A-F). Data are expressed using mean ± standard deviation.

Figure S2

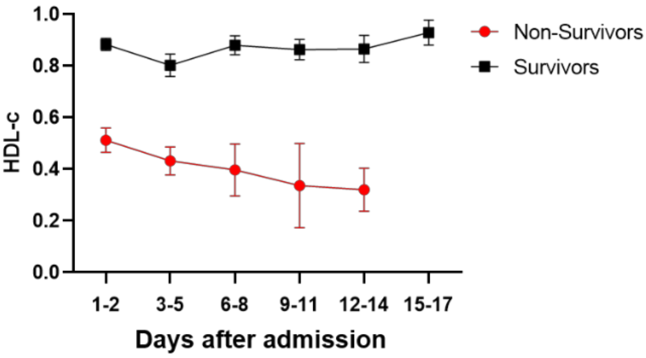

Figure S2. Dynamic changes of HDL-c levels in survivor and non-survivor SFTS patients (Blank, survivors; red, non-survivors)
